# Case Study on the Community Resilience Within Title VI Programs During COVID-19: A Qualitative Analysis

**DOI:** 10.1093/geroni/igab046.1795

**Published:** 2021-12-17

**Authors:** Abigail Bailey

**Affiliations:** Scripps Gerontology Center, Oxford, Ohio, United States

## Abstract

Health inequalities increased for Native Americans during the COVID-19 pandemic due to poor infrastructure, lack of electricity, health disparities, limited transportation, and rural location (Yellow Horse, 2021). Title VI programs-- aging network organizations that serve tribal elders--had to be resourceful to meet increased needs and restrictions on service delivery options. Qualitative data from the national 2020 Title VI Native American Aging Programs Survey illustrated the challenges faced and the resiliency of these organizations and their communities. Two rounds of thematic coding of 479 open-ended responses to the survey revealed that communication across organizations, a sense of shared mission, and sharing of resources allowed these agencies to provide more services in innovative ways. Challenges included limited funding, regulatory barriers, and staff burnout. A video presentation by a Title VI program director will provide context for the results of the survey.

